# Profiling cancer metabolism at the ‘omic’ level: a last resort or the next frontier?

**DOI:** 10.1186/s40170-016-0144-x

**Published:** 2016-03-21

**Authors:** Susan J. Gelman, Gary J. Patti

**Affiliations:** 1 Department of Chemistry, Washington University, St. Louis, MO 63130 USA; 2 Department of Medicine, Washington University School of Medicine, St. Louis, MO 63110 USA

When profiling metabolites, there are two general experimental paradigms: untargeted studies at the omic scale and targeted studies which usually focus on a much smaller subset of compounds. Although untargeted studies have become increasingly fashionable and are often perceived to be the cutting edge, it is important to recognize that they have limitations and are not always the better experiment. In theory, an untargeted analysis profiles all of the same metabolites as a targeted analysis, plus more. So why is more not always better?

We will frame our consideration in the words of pioneering computer scientist Alan Perlis: “Fools ignore complexity. Pragmatists suffer it. Some can avoid it. Geniuses remove it.” [1].

## “Fools ignore complexity.”

Indeed, untargeted metabolomic datasets are exceedingly complex. When applying a mass spectrometry-based platform, biological samples typically generate tens of thousands of signals per experiment. Even with state-of-the-art technologies, the majority of these signals cannot be annotated. Some signals are challenging to annotate because they are experimental artifacts, while others correspond to metabolites whose structure, function, and pathway remain unknown [2]. Without annotation, it is challenging to ascribe global meaning to the datasets. For example, it is precarious to compare the global metabolism of two samples on the basis of the percentage of signals changing because this percentage is highly dependent upon the number of artifacts.

## “Pragmatists suffer it.”

Despite its challenges, untargeted metabolomics is the workflow of choice for some research applications, such as identifying biomarkers of disease. In these applications, researchers do not have a biochemical hypothesis prior to beginning the analysis and generally only attempt to identify metabolomic signals that are potentially diagnostic of the condition being studied. A convincing example of the power of untargeted metabolomics to identify serum markers of non-small-cell lung cancer was recently published in the *Journal of Clinical Oncology*. Wikoff et al. found that the concentration of diacetylspermine increases approximately twofold in the blood collected from patients 6 months before diagnosis [3]. Untargeted metabolomics is also well suited for other types of unbiased screening applications. In *Cancer and Metabolism*, for instance, Gelman et al. reported an application of untargeted metabolomics to find metabolic products of the oncometabolite 2-hydroxyglutarate [4].

## “Some can avoid it.”

**Fig. 1:**
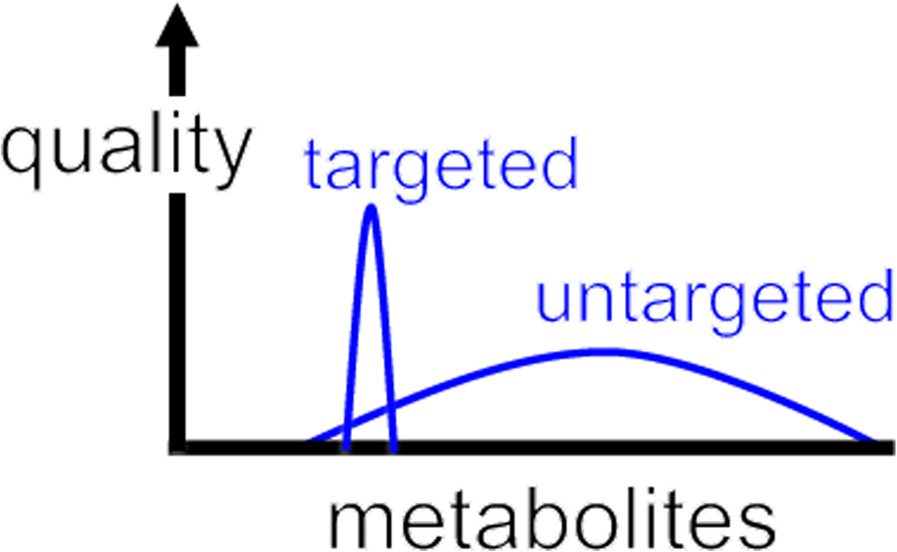
Schematic of tradeoff between number of metabolites detected and the quality at which they are measured in mass spectrometry-based metabolomics

The targeted analysis of metabolites provides data that are simpler to interpret compared to data from untargeted studies. Additionally, there is a tradeoff between the number of analytes measured by mass spectrometry and the quality of the measurement (Fig. 1). Untargeted metabolomics provides data on a larger number of compounds, but the data are less quantitatively reliable. Thus, if an investigator has a research question that can be answered with a targeted analysis, that is the recommended experiment. As a brief anecdotal example, we recently performed untargeted metabolomics in collaboration with another group at Washington University. After spending nearly a year running samples and performing data analysis, we discovered that our collaborators’ question could be answered directly by measuring a single metabolite. Instead of collaborating, our colleagues realized that they could more reliably measure the metabolite of interest by using a relatively inexpensive immunoassay in their own laboratory. As this account highlights, more is not always better. In their *Cancer and Metabolism* review, Salamanca-Cardona et al. discuss some other examples of when more is not better. They highlight how “targeted” hyperpolarized magnetic resonance imaging technologies can provide specific insights into cancer metabolism in vivo (such as pH, redox state, and tumor necrosis) [5].

## “Geniuses remove it.”

Great progress has been made over the last decade to improve the robustness of the mass spectrometry-based metabolomic platform and to facilitate the associated data processing. Instruments are becoming more quantitatively reliable, databases are expanding, software is advancing, computational approaches to assess flux are evolving, and informatic strategies to integrate gene expression data are being developed. Some of these advances are reviewed in *Cancer and Metabolism* by Markert et al. in “Mathematical Models of Cancer Metabolism” and in “Integration of Omics: More than the Sum of its Parts” by Buescher et al. [6, 7]. The objective is not only to reduce complexity but also to increase accessibility so that integrated omic experiments can be performed by scientists without extensive technical expertise.

This leaves us with an interesting question: should untargeted metabolomics be reserved as a last resort when no other experimental approaches can solve the problem of interest? We suggest that the answer is yes. However, the problems that metabolomic technologies uniquely position us to solve are of exceptional importance to the field of cancer metabolism and, in this sense, represent the next frontier.
